# Analysis of retinal and choroidal characteristics in patients with early diabetic retinopathy using WSS-OCTA

**DOI:** 10.3389/fendo.2023.1184717

**Published:** 2023-05-24

**Authors:** Zhihao Qi, Yuanyuan Si, Feng Feng, Jing Zhu, Xuepeng Yang, Wenjuan Wang, Yuting Zhang, Yan Cui

**Affiliations:** ^1^ Department of Ophthalmology, Qilu Hospital of Shandong University, Jinan, China; ^2^ Cheeloo College of Medicine, Shandong University, Jinan, China; ^3^ Department of Nephrology, Qilu Hospital of Shandong University, Jinan, China

**Keywords:** type 2 diabetes mellitus, early-diabetic retinopathy, widefield swept-source optical coherence tomography angiography, mid-large choroidal vessel, physiological indices, midperipheral fundus

## Abstract

**Introduction:**

Diabetic retinopathy (DR) is one of the most common and destructive microvascular complications of DM, and has become a major cause of irreversible visual impairment. The purpose of this study was to evaluate the changes in fundus microcirculation in non-diabetic retinopathy (NDR) and mild non-proliferative diabetic retinopathy (NPDR) in patients with type 2 diabetic mellitus (T2DM) using widefield swept-source optical coherence tomography angiography (WSSOCTA), and to investigate the correlation with laboratory indices of T2DM.

**Methods:**

Eighty nine, 58 and 28 eyes were included in the NDR, NPDR and Control groups, respectively, were enrolled in this study. The 12mm×12mm fundus images obtained by WSS-OCTA were divided into 9 regions (supratemporal, ST; temporal, T; inferotemporal, IT; superior, S; central macular area, C; inferior, I; supranasal, SN; nasal, N; inferonasal, IN) to evaluate changes in vessel density (VD) of the superficial capillary plexus (SCP), deep capillary plexus (DCP), choriocapillaris, and mid-large choroidal vessel (MLCV), as well as changes in inner retinal thickness (IRT), outer retinal thickness (ORT), and choroidal thickness (CT). Results: Compared with control group, MLCV VD (I, N, IN) was significantly decreased in NDR group, SCP VD (IT, C, I) and DCP VD (T, IT, I) were significantly decreased in NPDR group. In NPDR group, DCP VD (IT) was significantly decreased compared with that in NDR group. Compared with control group, CT (ST, T, IT, S, SN, IN) was significantly declined in NDR group, and IRT (ST, IT) and ORT (ST, N) were significantly increased in NPDR group. In NPDR group, IRT (ST) and ORT (T, S) were significantly increased compared with NDR group. Correlation analysis showed that age, body mass index, fasting blood glucose, fasting insulin, fasting C-peptide, and estimated glomerular filtration rate in T2DM patients were statistically correlated with retinal and choroidal thickness/VD.

**Discussion:**

Structural and blood flow changes in the choroid occur before the onset of DR and precede changes in the retinal microcirculation, and MLCV thickness/VD is a more sensitive imaging biomarker for the clinical detection of DR. WSS-OCTA enables large-scale non-invasive visual screening and follow-up of the retinal and choroidal vasculature in DR patients, providing a new strategy for the prevention and monitoring of DR in patients with T2DM.

## Introduction

Diabetes mellitus (DM), a metabolic disease characterized by chronic elevated blood glucose levels, can cause progressive damage to microvasculature, large vessels and nerves throughout the body (1, 2). Diabetic retinopathy (DR) is one of the most common and destructive microvascular complications of DM, and has become a major cause of irreversible visual impairment (3–5). Clinically, DR can be divided into two main stages: non-proliferative diabetic retinopathy (NPDR) and proliferative diabetic retinopathy (PDR). Damage to the retinal capillaries worsens as the disease progresses (6).

DM-induced vascular dysfunction eventually leads to tissue injury and degeneration, and thus the retinal changes in DR have always been of interest. It seems logical to conclude that DM also affects the choroidal vasculature (7). Retinal capillary abnormalities can be detected in many ways, but few detection devices can directly quantify the status of the mid-large choroidal vasculature (MLCV). Previous studies have used pulsatile ocular blood flow (POBF), choroidal vascularity index (CVI), and other indirect indicators of choroidal blood flow (8, 9). However, studies on MLCV in DR patients are still limited. Therefore, to investigate the relationship between choroidal blood flow changes and DR, an ancillary test that is non-invasive, visual, and directly measures choroidal perfusion may be helpful.

As an imaging technique with the advantages of speed, safety, high resolution and non-invasiveness, optical coherence tomography angiography (OCTA) has been widely used in clinical practice in recent years. It has a built-in quantitative analysis index to measure vessel morphology and density (10, 11). In addition, swept-source OCTA (SS-OCTA) can scan and generate 12mm×12mm images of the superficial, deep and choroidal retinal capillary layers, allowing perfusion studies of the choroidal matrix and lumen (12, 13). This study directly quantifies MLCV using widefield SS-OCTA (WSS-OCTA). With its unique built-in algorithm, WSS-OCTA has the ability to evaluate fundus abnormalities over a larger area, which may provide new insight into DR by correlating the vascular arc region in the central and mid-peripheral fundus.

PDR with severe visual impairment has generally received more attention from researchers than NPDR, which has seen little intervention. Recent studies have shown that about 50% of people with DM have mild visual problems (14). A more in-depth study of the fundus of diabetic patients is essential to monitor preclinical and early DR. Previous studies of DR using OCTA were limited to a 3mm×3mm or 6mm×6mm fundus area. However, most of the histopathologic changes in diabetic chorioretinopathy (DC) were found in the midperipheral area (7), so there was an urgent need to study DR on a larger scale (15–17). By performing thickness/vessel density (VD) analyses with a scan area of 12mm×12mm, we systematically described the circulatory and structural characteristics of the central and midperipheral retina and choroid in preclinical/early-stage DR. On this basis, the correlation between VD/thickness of each fundus layer and clinical-physiological indices in T2DM patients was also investigated. The aim of this study is to detect fundus changes and damage in T2DM patients earlier and to provide guidance for clinical prevention and monitoring.

## Materials and methods

### Study participants

According to the diagnostic criteria of T2DM in the *Clinical guidelines for prevention and treatment of type 2 diabetes mellitus in the elderly in China* (2022 edition) (18), 93 patients with T2DM and 20 healthy subjects of similar age were enrolled in Qilu Hospital of Shandong University from November 2021 to March 2022. All subjects underwent comprehensive ophthalmologic examinations, including best corrected visual acuity (BCVA), axial length (AL), spherical equivalent, intraocular pressure (IOP), slit lamp, high resolution optical coherence tomography (OCT), fundus color photography and WSS-OCTA. And we collected subjects’ demographic data (sex, age, body mass index (BMI), history of T2DM) and clinical laboratory indices (fasting blood glucose, FBG; fasting insulin, FINS; fasting C-peptide, FCP; glycosylated hemoglobin type A1c, HbA1c; estimated glomerular filtration rate, eGFR).

Inclusion criteria for subjects were as follows: 1) age ≥ 18 years old; 2) BCVA ≥ z1.0log-MAR, spherical equivalent ≤ ± 1.50D, IOP ≤ 21.00 mmHg; 3) clear optical media. Exclusion criteria were as follows: 1) AL ≥ 26mm or AL ≤ 21mm; 2) ocular trauma or ocular surgery; 3) treatment history of panretinal laser photocoagulation or anti-vascular endothelial growth factor therapy; 4) ocular complications, such as keratitis, conjunctivitis, glaucoma, vitreous opacity, retinal detachment, macular edema, anterior macular membrane, pigmented retinitis, retinal arteriovenous occlusion and etc.; 5) the low quality of binocular WSS-OCTA image and the patients with poor compliance; 6) medication history of anticoagulant, lipid-lowering and antioxidant drugs; 7) infectious diseases; 8) pregnant. According to the *Clinical Practice Guidelines on the Management of Nonproliferative and Proliferative Diabetic Retinopathy without Diabetic Macular Edema* proposed by the American Society of Retina Specialists in 2020 (19), the patients with T2DM were divided into NDR group and NPDR group. Due to the inconsistency of binocular clinical manifestations in some individuals with T2DM, we only included eyes with more severe clinical diagnosis in these individuals. Finally, 89, 58 and 28 eyes were included in the NDR, NPDR and control groups, respectively. Written informed consent was obtained before examinations (version 1.0 dint 20210605001). This study adhered to the tenets of the Declaration of Helsinki and was approved by the Ethics Committee of Qilu Hospital (KYLL-202111-024-1).

### WSS-OCTA device parameters and observation indicators

**Figure 1:**
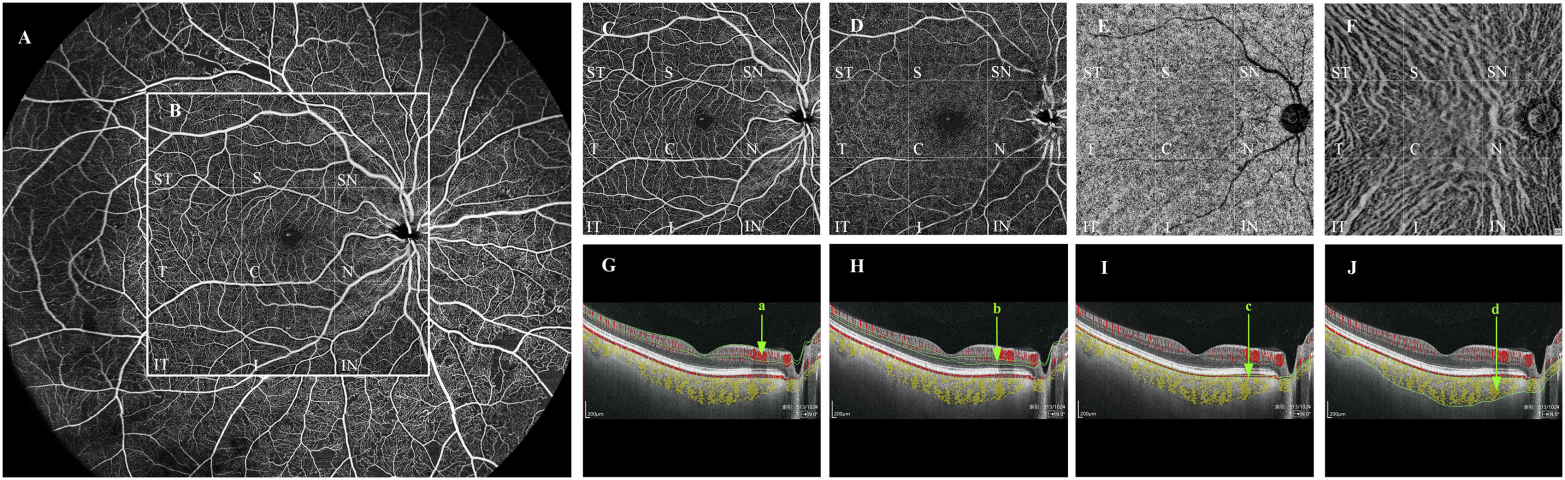
Quantification of vessel density in each vascular layer of the fundus by WSS-OCTA. Taking the right fundus vascular image obtained by WSS-OCTA in the 24 mm × 20 mm region as an example **(A)**, we selected the binarized images of the fundus in the 12mm×12mm area for VD quantitative analysis, and divided them by the built-in software into nine 4mm×4mm regions (ST, T, IT, S, C, I, SN, N, and IN **(B)**). Each cross-sectional image obtained by WSS-OCTA scanning was automatically sectioned by the built-in software into four vascular layers, namely, SCP, DCP, CC, and MLCV **(C–F)**, and divided into the above nine 4mm×4mm regions, the VD of each region in each vascular layer was quantified. **(G–J)** are imaging tomography at the level of the macular fovea; the areas within the green line (a–d) are SCP **(C)**, DCP **(D)**, CC **(E)**, and MLCV **(F)**, respectively. VD, vessel density; ST, supratemporal; T, temporal; IT, inferotemporal; S, superior; C, central macular area; I, inferior; SN, supranasal; N, nasal; IN, inferonasal; SCP, superficial capillary plexus; DCP, deep capillary plexus; CC, choriocapillary; MLCV, mid-to-large choroidal vessel.

The WSS-OCTA device (YG100K Yalkaid, TowardPi Medical Technology Co., Ltd., Beijing, China) uses a wide-field laser source for imaging with a scan frequency of 100 kHz, a depth of 6 mm, an area of 24 mm × 20 mm, and an image resolution of 3.8 μm (**Figure 1A**).

**Figure 2:**
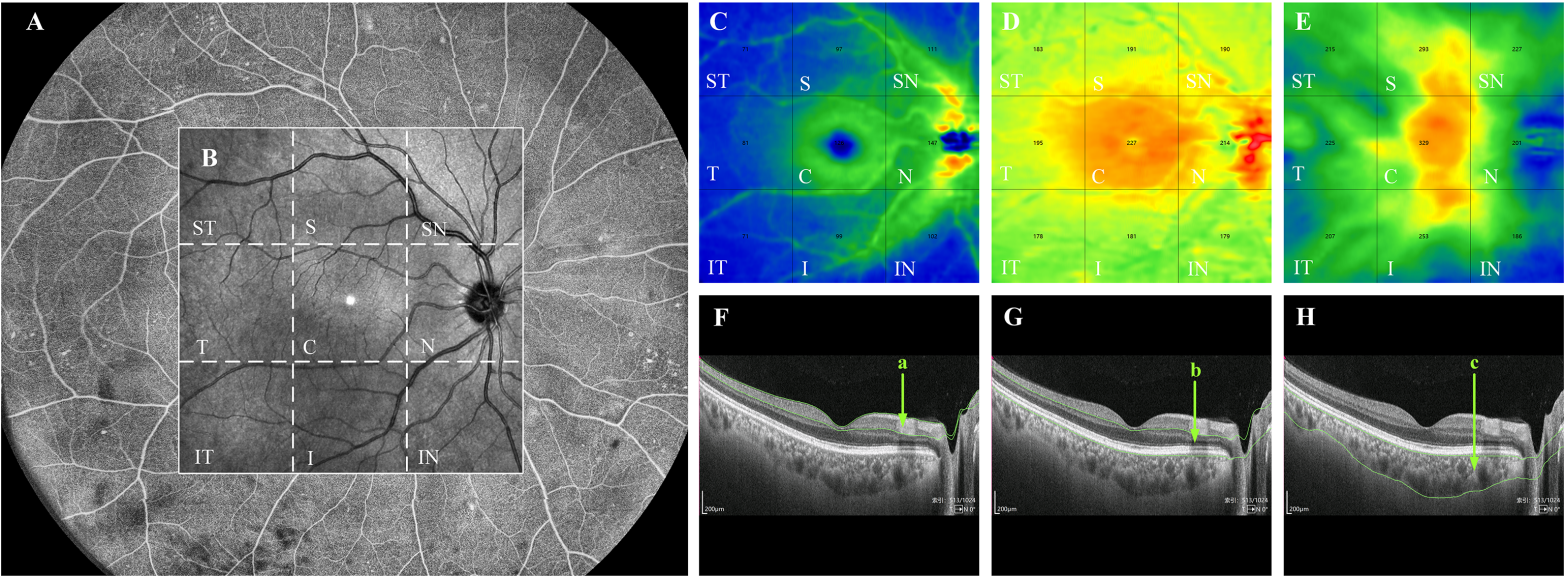
Quantification of thickness in each layer of the fundus by WSS-OCTA. WSS-OCTA can scan fundus images in the range of 24mm×20mm **(A)**. In this study, fundus image data in the range of 12mm×12mm were selected for quantitative thickness analysis **(B)**. Each cross-sectional image obtained by WSS-OCTA scanning was automatically sectioned by the built-in software into three thickness topographic maps, namely, IRL, ORL and choroid **(C–E)**, each image is divided by the built-in software into nine regions, namely, ST, S, SN, T, C, N, IT, I, and IN regions. The thickness of each region in each layer is quantified. **(F–H)** are the imaging tomography at the level of the macular fovea, where the distance between the green lines in (a–c) indicate the IRT, ORT, and CT, respectively. IRL, inner retinal layer; ORL outer retinal layer; ST, supratemporal; T, temporal; IT, inferotemporal; S, superior; C, central macular area; I, inferior; SN, supranasal; N, nasal; IN, inferonasal; IRT, inner retinal thickness; ORT, outer retinal thickness; CT, choroidal thickness.

The device has real-time high-frequency eye-tracking technology and motion correction algorithms to avoid image artifacts caused by eye movements. Scanning the fundus in B-scan mode, we selected images in the range of 12 mm × 12 mm area (**Figure 1B**), and the device automatically identified four vascular layers (**Figures 1C–F**), including the SCP from the internal limiting membrane (ILM) to 9 μm above the inner plexiform layer (IPL), the DCP from the interface of 9 μm above the IPL to 9μm below the outer plexiform layer (OPL), the CC 29 μm below the Bruch’s membrane (BM), and the MLCV 29 μm beneath the BM to the choroid-sclera interface (CSI) (**Figures 1G–J**). VD, the ratio of the vascularized area to the total image area, was automatically quantified by a high-order moment amplitude decorrelation angiography algorithm. And from the 24 mm × 20 mm area of the Enface images (**Figure 2A**), the 12 mm × 12 mm area was selected for thickness analysis (**Figure 2B**). The built-in software automatically generated topographic maps of the thickness of the inner retinal layer (IRL), the outer retinal layer (ORL) and the choroidal layer (**Figures 2C–E**). The inner retinal thickness (IRT) is the distance from the ILM to the IPL, the outer retinal thickness (ORT) is the distance from the IPL to the BM, and the choroidal thickness (CT) is the distance from the BM to the CSI (**Figures 2F–H**).

The 12mm × 12mm images of each layer of the fundus were automatically divided into nine 4mm × 4mm regions: supratemporal (ST), temporal (T), inferotemporal (IT), superior (S), central macular area (C), inferior (I), supranasal (SN), nasal (N), inferonasal (IN). The VD/thickness of each region at each layer was quantified. Each eye was scanned twice and the results were averaged. All WSS-OCTA examinations were performed by one trained operator.

### Statistical analysis

In this study, we mainly used binocular data, which do not satisfy the assumption of data independence due to the existence of hierarchical structure in individuals, eye categories, layers and regions, and the strong correlation of binocular data in the same individual. Therefore, we used a generalized linear mixed-effects model for the between-group variability analysis of retinal and choroidal thickness/VD data. This analysis was performed using the lmer and glmmTMB functions in R. Statistical significance was considered at P<0.05. Analysis of the correlation between laboratory indices and fundus indicators in T2DM subjects was performed by a linear mixed-effects model, and this analysis was implemented by the glmer function in R. Statistical significance was considered at P<0.05.

## Results

### Demographics characteristics and clinical laboratory indices

Demographic and laboratory characteristics of the study population are shown in **Table 1**. A total of 175 eyes were included in our study; 89, 58 and 28 eyes were included in the NDR, NPDR, and control groups, respectively. There were no significant differences in age, BMI and FCP among the three groups. Compared with the control group, the NDR and NPDR groups showed statistically significant differences in FBG, FINS, HbA1c and eGFR.

**Table 1:** Demographics characteristics and clinical physiological indexes of each group.

	Control	NDR	NPDR	P_1_	P_2_	P_3_
Patients (male)	20 (9)	53 (28)	40 (24)	/	/	/
Eyes	28	89	58	/	/	/
Age (years)	59.70 ± 6.95	58.55 ± 10.38	58.00 ± 10.15	0.80	0.97	0.85
BMI (kg/m^2^)	24.90 ± 2.93	25.63 ± 3.71	25.99 ± 3.25	0.45	0.21	0.48
FBG (mmol/L)	5.00 ± 0.66	8.58 ± 3.04	9.24 ± 3.30	<0.01**	<0.01**	0.94
FINS (μ IU/mL)	5.16 ± 2.84	14.49 ± 14.61	15.75 ± 13.98	<0.01**	<0.01**	0.93
FCP (ng/mL)	1.28 ± 0.96	1.24 ± 0.91	1.20 ± 0.80	0.69	0.67	0.48
HbA1c (%)	5.98 ± 0.60	8.83 ± 1.83	8.69 ± 1.53	<0.01**	<0.01**	0.31
eGFR (mL/min/1.73m^2^)	95.31 ± 15.16	115.55 ± 44.43	109.75 ± 31.01	<0.01**	0.02*	0.97

Statistically significant values are shown with */**P<0.05 is marked by *P<0.01 is marked by **P_1_: Control vs. NDR; P_2_: Control vs. NPDR; P_3_: NDR vs. NPDR.

Control, healthy subjects; NDR, non-diabetic retinopathy; NPDR, non-proliferative diabetic retinopathy; FBG, fasting blood-glucose; FINS, fasting insulin; FCP, fasting C-peptide; HbA1c, glycosylated hemoglobin type A1c; eGFR, estimated glomerular filtration rate.

### Thickness analysis of retina and choroid

**Figure 3:**
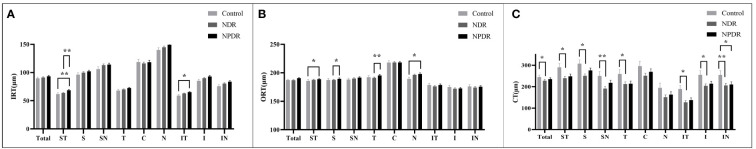
Thickness of retina and choroid in nine 4mm×4mm regions. IRT **(A)**, ORT **(B)** and CT **(C)** are quantified and compared between groups. Statistically significant values are indicated with *, *P< 0.05, **P< 0.01. Control, healthy subjects; NDR, non-diabetic retinopathy; NPDR, non-proliferative diabetic retinopathy; IRT, inner retinal thickness; ORT, outer retinal thickness; CT, choroidal thickness.

**Figure 4:**
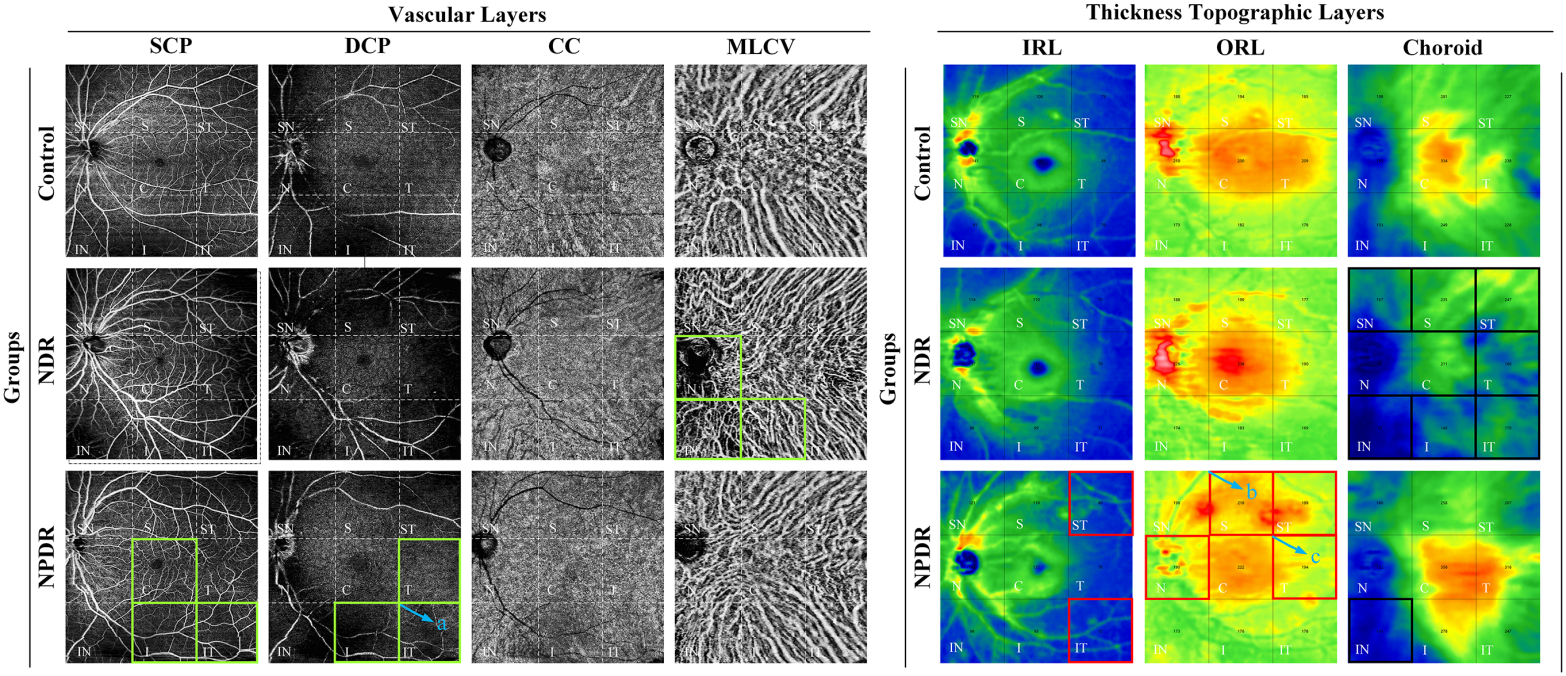
Summary of VD/thickness differences in 9 regions of each layer of retina and choroid. Regions with decreased VD are indicated by the green boxes. NPDR vs. control: SCP VD in C, I, and IT regions, DCP VD in T, I, and IT regions decreased in the NPDR group. NDR vs. control: MLCV VD decreased in N, IN, and I regions in the NDR group. The IT region where DCP VD was decreased in the NPDR group compared to the NDR group is shown in the green box in **(a)**. Regions of increased thickness are indicated by the red boxes. NPDR vs. control: IRT in ST and IT regions, ORT in N and ST regions increased in NPDR group. NPDR vs NDR: ORT in S **(b)** and T **(c)** regions increased in the NPDR group. Regions with decreased thickness are indicated by the black boxes, which CT decreased in ST, T, IT, S, SN, and IN regions decreased in the NDR group and IN regions decreased in the NPDR group compared to control. Control, healthy subjects; NDR, non-diabetic retinopathy; NPDR, non-proliferative diabetic retinopathy; VD, vessel density; SCP, superficial capillary plexus; DCP, deep capillary plexus; CC, choriocapillaris; MLCV, mid-to-large choroidal vessel; IRL, inner rtinal layer, ORL, outer retinal layer, IRT, inner retinal thickness; ORT, outer retinal thickness; CT, choroidal thickness, ST, supratemporal; T, temporal; IT, inferotemporal; S, superior; C, central macular area; I, inferior; SN, supranasal; N, nasal; IN, inferonasal.

In the total area of 12mm×12mm fundus, there was no significant difference in IRT and ORT among the three groups (**Figures 3A, B**). And compared with the control group, a significant decrease in CT was observed in the NDR group (**Figure 3C**). Further analysis showed that there was no significant difference in IRT and ORT between the control and NDR groups in all 4mm×4mm regions. However, IRT was significantly increased in ST and IT regions in the NPDR group compared to the control group (**Figure 3A**). ORT in NPDR group was significantly increased in ST and N regions compared with control (**Figure 3B**). Furthermore, the NPDR group showed significantly increased IRT in ST region, ORT in T and S regions compared to the NDR group (**Figures 3A, B**). Compared with the control group, CT in ST, T, IT, S, I, SN, and IN regions was significantly decreased in the NDR group, and CT in IN region was significantly decreased in the NPDR group (**Figure 3C**). Details of thickness changes in each area of each group are shown in **Table 2**; **Figure 4**.

**Table 2:** Thickness differences of fundus in each layer and region between groups.

Layer	Region	Control	NDR	NPDR	P_1_	P_2_	P_3_
IRT	Total	92.00 ± 30.97	92.75 ± 28.48	95.32 ± 27.91	0.691	0.174	0.191
	ST	61.71 ± 6.13	62.92 ± 7.24	68.22 ± 9.12	0.337	0.001**	0.000**
	S	96.86 ± 9.93	97.90 ± 12.07	101.33 ± 13.75	0.525	0.197	0.217
	SN	109.61 ± 12.17	111.04 ± 14.94	113.50 ± 15.75	0.736	0.934	0.518
	T	68.36 ± 7.77	69.38 ± 7.14	72.47 ± 10.03	0.348	0.057	0.137
	C	119.43 ± 36.51	116.88 ± 10.98	118.12 ± 12.02	0.601	0.817	0.845
	N	145.04 ± 15.71	145.29 ± 16.28	144.29 ± 16.65	0.889	0.760	0.477
	IT	60.50 ± 4.93	62.17 ± 5.64	64.83 ± 8.87	0.198	0.015*	0.090
	I	87.79 ± 8.92	89.27 ± 8.31	91.91 ± 13.18	0.438	0.113	0.181
	IN	78.75 ± 9.25	79.87 ± 9.44	83.22 ± 9.81	0.202	0.298	0.114
ORT	Total	187.15 ± 17.12	187.08 ± 16.64	190.94 ± 17.40	0.893	0.116	0.312
	ST	184.46 ± 9.69	186.69 ± 9.39	189.22 ± 8.75	0.275	0.048*	0.103
	S	187.04 ± 7.87	186.11 ± 8.83	190.7 ± 29.95	0.867	0.051	0.048*
	SN	187.93 ± 8.11	188.93 ± 8.89	192.93 ± 11.03	0.424	0.063	0.090
	T	191.32 ± 11.31	190.62 ± 10.53	197.07 ± 11.73	0.868	0.058	0.014*
	C	217.75 ± 11.32	217.03 ± 10.85	220.95 ± 11.36	0.835	0.550	0.547
	N	193.82 ± 8.08	196.25 ± 11.64	199.83 ± 14.14	0.368	0.043*	0.131
	IT	175.93 ± 15.88	175.78 ± 9.54	178.10 ± 9.02	0.852	0.495	0.208
	I	172.32 ± 13.24	170.62 ± 8.72	173.88 ± 9.22	0.544	0.896	0.277
	IN	173.75 ± 12.89	171.71 ± 9.38	175.72 ± 9.71	0.359	0.935	0.166
CT	Total	241.04 ± 96.81	207.81 ± 90.36	214.84 ± 75.26	0.015*	0.084	0.365
	ST	274.07 ± 68.13	237.02 ± 75.85	249.26 ± 63.45	0.032*	0.118	0.433
	S	294.93 ± 71.99	255.15 ± 92.76	270.05 ± 66.82	0.026*	0.192	0.252
	SN	240.79 ± 80.01	197.96 ± 87.52	208.00 ± 59.45	0.008**	0.113	0.158
	T	248.14 ± 85.95	213.47 ± 77.05	217.90 ± 61.80	0.040*	0.088	0.809
	C	281.89 ± 94.50	255.64 ± 98.00	269.69 ± 78.26	0.094	0.357	0.406
	N	178.00 ± 95.57	154.53 ± 83.20	160.69 ± 64.81	0.085	0.219	0.675
	IT	243.86 ± 99.50	213.66 ± 74.18	214.12 ± 58.45	0.049*	0.131	0.644
	I	238.11 ± 97.68	210.08 ± 77.94	212.45 ± 53.54	0.032*	0.083	0.791
	IN	169.57 ± 103.18	132.80 ± 63.21	131.45 ± 42.81	0.009**	0.025*	0.694

Statistically significant values are shown with */**P<0.05 is marked by* P<0.01 is marked by**. P_1_:Control vs. NDR; P_2_:Control vs. NPDR; P_3_:NDR vs. NPDR. Control, healthy subjects; NDR, non-diabetic retinopathy; NPDR, non-proliferative diabetic retinopathy; IRT, inner retinal thickness; ORT, outer retinal thickness; CT, choroidal thickness; ST, supratemporal; T, temporal; IT, inferotemporal; S, superior; C, central macular area; I, inferior; SN, supranasal; N, nasal; IN, inferonasal.

### VD analysis of retina and choroid

**Figure 5:**
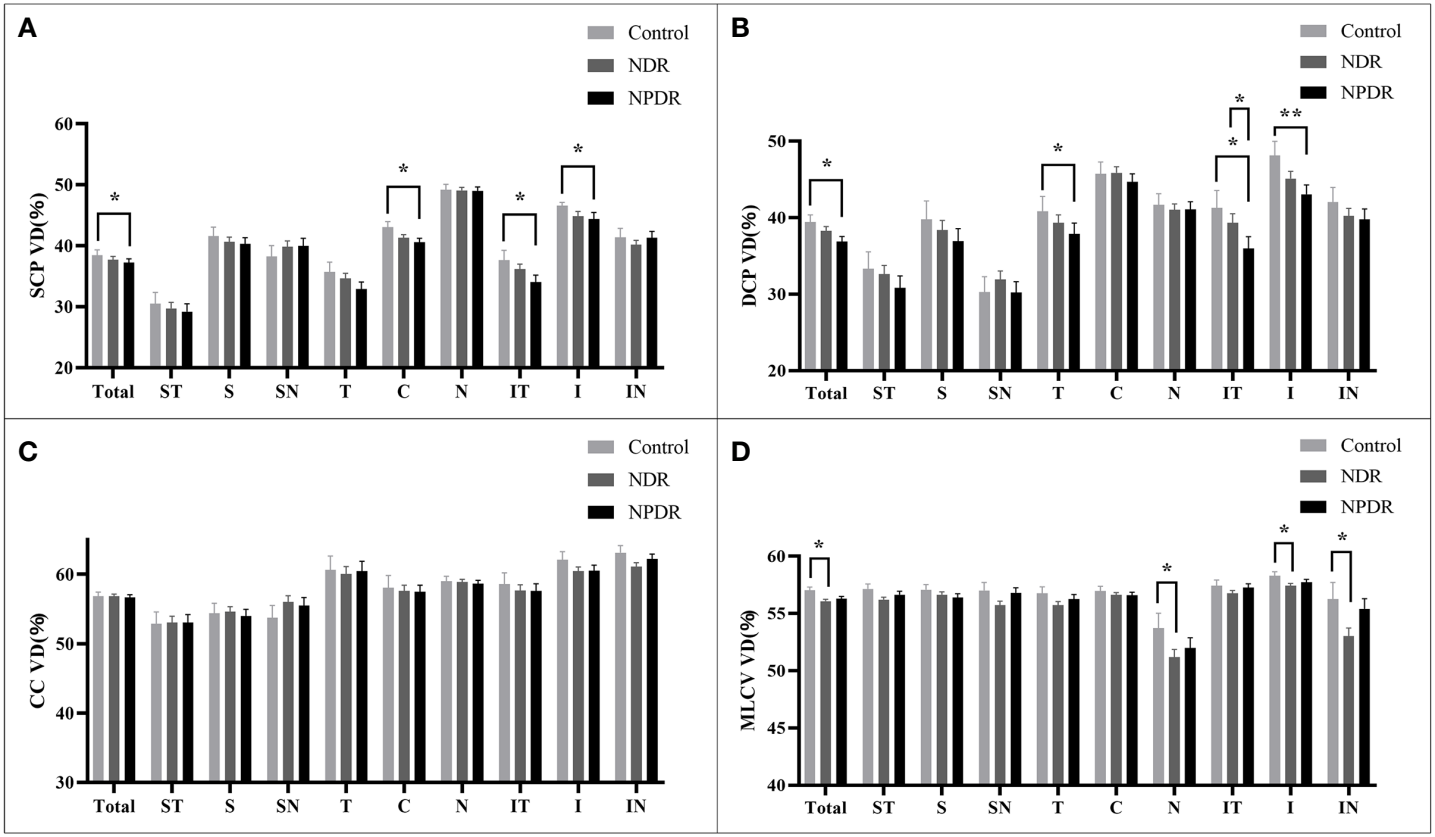
VD of retina and choroid in nine 4mm×4mm regions. SCP VD **(A)**, DCP VD **(B)**, CC VD **(C)** and MLCV VD **(D)** are quantified and compared between groups. Statistically significant values are indicated with *, *P< 0.05, **P< 0.01. Control, healthy subjects; NDR, non-diabetic retinopathy; NPDR, non-proliferative diabetic retinopathy, VD, vessel density; SCP, superficial capillary plexus; DCP, deep capillary plexus; CC, choriocapillaris; MLCV, mid-to-large choroidal vessel.

In the total area of 12mm×12mm fundus, the VD of SCP and DCP in NPDR group was significantly lower than that in control group (**Figures 5A, B**). In addition, decreased VD of MLCV was observed in the NDR group compared with control group (**Figure 5D**).

In all 4mm×4mm regions, VD of SCP and DCP was not significantly different in the NDR group compared with the control group. Compared with the control group, the VD of IT, C, I regions in SCP and T, IT, I regions in DCP were significantly reduced in the NPDR group (**Figures 5A, B**). In addition, VD in the IT region of the DCP was significantly declined in NPDR group compared to the NDR group.

There was no significant difference in VD of the CC layer between the three groups (**Figure 5C**).

Compared to the control group, VD in MLCV layer was significantly decreased in the I, N, and IN regions in the NDR group, but did not differ in all regions in the NPDR group (**Figure 5D**). Details of VD changes in each area of each group are shown in **Table 3** and **Figure 4**.

**Table 3:** VD differences of fundus in each layer and region between groups.

Layer	Region	Control	NDR	NPDR	P_1_	P_2_	P_3_
SCP	Total	40.41 ± 8.03	39.60 ± 8.14	39.08 ± 8.06	0.181	0.037*	0.600
	ST	30.50 ± 7.63	29.73 ± 8.12	29.19 ± 6.91	0.591	0.465	0.724
	S	41.54 ± 6.65	40.65 ± 5.74	40.31 ± 5.69	0.727	0.576	0.809
	SN	38.21 ± 9.34	39.83 ± 6.78	39.97 ± 7.50	0.254	0.283	0.952
	T	35.71 ± 6.15	34.63 ± 7.03	32.93 ± 5.34	0.427	0.069	0.187
	C	43.00 ± 2.93	41.33 ± 4.30	40.55 ± 4.41	0.092	0.023*	0.434
	N	49.18 ± 2.72	49.09 ± 4.18	49.02 ± 3.17	0.895	0.845	0.869
	IT	37.61 ± 6.83	36.13 ± 6.93	34.05 ± 6.35	0.355	0.031*	0.085
	I	46.54 ± 4.31	44.87 ± 4.77	44.34 ± 5.27	0.121	0.044*	0.573
	IN	41.39 ± 4.94	40.13 ± 6.49	41.33 ± 5.34	0.287	0.939	0.208
DCP	Total	40.39 ± 9.79	39.24 ± 9.39	37.63 ± 9.32	0.428	0.045*	0.142
	ST	33.32 ± 9.45	32.60 ± 8.85	30.84 ± 9.54	0.746	0.207	0.191
	S	39.75 ± 9.59	38.39 ± 9.07	36.93 ± 9.94	0.647	0.329	0.497
	SN	30.29 ± 12.26	31.93 ± 10.23	30.43 ± 10.12	0.372	0.794	0.347
	T	40.43 ± 8.94	39.21 ± 7.98	36.67 ± 7.74	0.587	0.036*	0.080
	C	46.64 ± 6.34	45.35 ± 6.12	43.84 ± 6.16	0.393	0.108	0.271
	N	41.68 ± 3.94	41.03 ± 6.90	41.09 ± 5.66	0.589	0.726	0.765
	IT	41.25 ± 8.94	39.34 ± 8.68	35.98 ± 8.18	0.390	0.022*	0.034*
	I	48.14 ± 6.37	45.12 ± 7.40	43.07 ± 8.19	0.070	0.003**	0.136
	IN	42.04 ± 6.06	40.21 ± 9.03	39.79 ± 6.91	0.227	0.208	0.755
CC	Total	58.06 ± 6.15	57.83 ± 5.71	57.70 ± 6.37	0.653	0.527	0.737
	ST	52.86 ± 6.28	53.06 ± 6.59	53.03 ± 7.20	0.843	0.943	0.870
	S	54.39 ± 5.59	54.60 ± 4.98	53.98 ± 6.01	0.737	0.927	0.599
	SN	53.75 ± 9.16	56.00 ± 5.28	55.47 ± 7.02	0.451	0.869	0.627
	T	60.64 ± 3.53	60.06 ± 3.48	60.47 ± 5.70	0.561	0.587	0.955
	C	58.04 ± 2.74	57.61 ± 6.81	57.45 ± 4.22	0.674	0.786	0.775
	N	59.00 ± 2.61	58.89 ± 3.11	58.64 ± 3.14	0.854	0.702	0.971
	IT	58.61 ± 5.92	57.66 ± 5.61	57.57 ± 6.34	0.462	0.438	0.913
	I	62.11 ± 3.38	60.46 ± 4.08	60.53 ± 5.20	0.071	0.098	0.861
	IN	63.11 ± 3.34	61.12 ± 3.96	62.19 ± 5.21	0.637	0.992	0.815
MLCV	Total	56.73 ± 2.31	55.48 ± 4.44	56.12 ± 2.95	0.018*	0.158	0.342
	ST	57.14 ± 1.48	56.20 ± 2.23	56.64 ± 1.51	0.051	0.239	0.361
	S	57.07 ± 1.41	56.65 ± 1.45	56.40 ± 2.71	0.332	0.152	0.228
	SN	57.00 ± 1.59	55.72 ± 3.73	56.81 ± 1.76	0.123	0.602	0.208
	T	56.75 ± 2.01	55.73 ± 3.24	56.26 ± 1.93	0.105	0.361	0.545
	C	56.96 ± 1.69	56.62 ± 1.43	56.59 ± 1.62	0.326	0.469	0.406
	N	53.71 ± 3.61	51.17 ± 6.50	51.98 ± 4.22	0.047*	0.190	0.601
	IT	57.43 ± 1.73	56.76 ± 2.40	57.26 ± 1.54	0.133	0.496	0.363
	I	58.29 ± 1.33	57.43 ± 1.85	57.72 ± 1.29	0.017*	0.100	0.665
	IN	56.25 ± 2.25	53.01 ± 7.75	55.41 ± 3.96	0.045*	0.441	0.064

Statistically significant values are shown with */**P<0.05 is marked by *P<0.01 is marked by **P_1_: Control vs. NDR; P_2_: Control vs. NPDR; P_3_: NDR vs. NPDR. The unit of VD is percentage (%). Control, healthy subjects; NDR, non-diabetic retinopathy; NPDR, non-proliferative diabetic retinopathy; VD, vessel density; SCP, superficial capillary plexus; DCP, deep capillary plexus; CC, choriocapillary; MLCV, mid-to-large choroidal vessel; ST, supratemporal; T, temporal; IT, inferotemporal; S, superior; C, central macular area; I, inferior; SN, supranasal; N, nasal; IN, inferonasal.

### Correlation analysis between laboratory indexes and thickness/VD in T2DM patients

We analyzed the correlations between thickness in each region at each layer of the fundus and laboratory indices in patients with T2DM. FBG was positively correlated with ORT in Total, ST, T, SN, and N regions. BMI had positive correlation with ORT in Total, ST, S, C, SN, and IN regions, as well as CT in ST region. eGFR was positively related with IRT in Total, S, C, and I regions, as well as CT in Total, I, N and IN regions. In addition, we found that age was negatively correlated with the thickness of all regions in each layer. FINS was related with CT of the Total area negatively. And FCP and HbA1c were not correlated with the thickness of each area of each layer. See **Table 4** for details.

**Table 4:** Correlation analysis between thickness and clinical physiological indexes in T2DM.

Layer	Region	Age		BMI		FBG		FINS		FCP		HbA1c		eGFR	
		ES	P	ES	P	ES	P	ES	P	ES	P	ES	P	ES	P
**IRT**	**Total**	-.263	.005**	-.199	.488	.212	.519	-.060	.390	-.471	.695	.572	.358	.068	.008**
	**ST**	-.119	.125	.187	.400	.220	.393	-.002	.978	.033	.969	.451	.354	.028	.163
	**T**	-.097	.228	.245	.280	.257	.984	-.034	.559	.004	.996	.163	.742	.028	.179
	**IT**	-.103	.082	-.008	.961	.296	.111	.021	.635	-.374	.574	.482	.482	.028	.075
	**S**	-.359	.002**	.453	.178	.380	.328	-.121	.157	.660	.639	.802	.275	.079	.010**
	**C**	-.298	.005**	.533	.077	.463	.178	-.065	.401	.510	.684	.796	.223	.093	.001**
	**I**	-.261	.003**	.166	.528	.233	.448	-.011	.866	.200	.860	.365	.533	.059	.012*
	**SN**	-.364	.009**	.255	.529	.186	.692	-.195	.055	-1.453	.396	.453	.609	.055	.137
	**N**	-.575	.000**	.063	.886	.450	.366	-.141	.202	-1.264	.493	.909	.336	.121	.002**
	**IN**	-.111	.245	.209	.436	.133	.665	.051	.457	-.157	.888	.719	.214	.036	.146
**ORT**	**Total**	-.244	.000**	.519	.004	.593	.003**	-.074	.089	.832	.276	.048	.903	.020	.219
	**ST**	-.290	.000**	.608	.012	.598	.034*	-.067	.285	1.648	.116	.204	.705	.014	.545
	**T**	-.295	.007**	.578	.065	.836	.020*	-.073	.363	1.830	.171	.664	.332	.028	.331
	**IT**	-.126	.207	.317	.250	.535	.078	-.047	.515	-1.083	.340	.451	.395	.039	.139
	**S**	-.177	.070	.582	.026	.388	.187	-.119	.076	1.264	.244	-.266	.640	.010	.699
	**C**	-.170	.142	.738	.015	.266	.427	-.034	.666	1.670	.184	-.214	.745	.036	.236
	**I**	-.224	.009**	.299	.220	.342	.221	-.084	.178	-.413	.691	.186	.727	.044	.049
	**SN**	-.240	.011*	.671	.012	.745	.015*	-.093	.175	.371	.749	.427	.471	.013	.610
	**N**	-.261	.015*	.481	.124	1.326	.000**	-.117	.146	.679	.617	1.037	.141	.030	.292
	**IN**	-.252	.008**	.610	.022	.460	.130	-.037	.592	-.170	.881	.316	.588	.030	.236
**CT**	**Total**	-3.060	.000**	2.403	.079	.381	.803	-.646	.023*	9.162	.095	.576	.833	.454	.000**
	**ST**	-2.486	.000**	3.767	.041*	1.492	.486	.100	.835	13.483	.087	-.345	.932	-.053	.762
	**T**	-2.187	.001**	2.170	.258	.341	.877	-.311	.528	11.195	.166	.728	.862	.124	.491
	**IT**	-1.869	.007**	2.547	.180	-.539	.797	-.736	.142	3.596	.646	-3.351	.360	.097	.602
	**S**	-3.338	.000**	2.895	.188	.264	.917	-.199	.726	9.647	.301	-1.152	.820	.133	.523
	**C**	-3.146	.000**	.743	.716	-1.063	.703	-.546	.380	9.808	.340	1.625	.759	.365	.107
	**I**	-3.061	.000**	2.871	.117	1.402	.506	-.264	.578	9.198	.238	2.766	.490	.406	.018
	**SN**	-2.327	.001**	3.709	.074	-1.950	.414	-.190	.723	14.913	.090	-1.512	.740	.210	.286
	**N**	-1.916	.008**	.853	.678	-2.228	.343	-.472	.370	12.394	.153	1.881	.674	.422	.026
	**IN**	-1.811	.000**	1.781	.230	-2.069	.226	-.174	.647	6.891	.277	.598	.584	.349	.010

Statistically significant values are shown with */**P<0.05 is marked by *P<0.01 is marked by **. ES: effect size (um). FBG, fasting blood-glucose; FINS, fasting insulin; FCP, fasting C-peptide; HbA1c, glycosylated hemoglobin type A1c; eGFR, estimated glomerular filtration rate; IRT, inner retinal thickness; ORT, outer retinal thickness; CT, choroidal thickness.

The correlations between VD in different regions of each layer and laboratory indices in T2DM patients were also analyzed. FBG was negatively correlated with VD of Total, S, SN, and N regions in DCP layer. BMI was related with VD of the C region in MLCV layer positively, but had negative relationship with VD of ST, S, and SN regions in SCP layer, Total, ST, S, and SN regions in DCP layer, and VD of S region in CC layer. eGFR was negatively correlated with VD of both N region in DCP layer and ST region in MLCV layer. FINS was positively correlated with VD of N region in SCP layer and negatively correlated with VD of regions containing I in DCP layer, ST in CC layer and S in MLCV layer. VD of SN in SCP layer, Total, ST, S and SN in DCP layer, S and SN in CC layer had negative relationship with FCP. Age was negatively correlated with VD of most regions in MLCV layer, while HbA1c was not related with VD in each region of each layer. See **Table 5** for details.

**Table 5:** Correlation analysis between VD and clinical physiological indexes in T2DM.

Layer	Region	Age		BMI		FBG		FINS		FCP		HbA1c		eGFR	
		ES	P	ES	P	ES	P	ES	P	ES	P	ES	P	ES	P
**SCP**	**Total**	-.007	.853	-.193	.087	-.123	.332	-.015	.554	-.756	.101	-.103	.663	.008	.438
	**ST**	.012	.844	-.508	.003**	-.320	.144	-.062	.157	-1.615	.033*	-.446	.258	.021	.170
	**T**	.069	.167	-.073	.615	-.151	.378	.027	.459	-.405	.529	-.062	.853	.000	.975
	**IT**	-.019	.703	.046	.751	-.041	.800	-.007	.860	.780	.211	-.205	.474	.001	.946
	**S**	-.001	.988	-.391	.004**	-.195	.233	-.047	.185	-1.729	.004	.098	.753	.014	.281
	**C**	.038	.307	-.044	.681	-.127	.309	-.005	.856	-.451	.329	.029	.902	-.010	.318
	**I**	-.053	.228	-.002	.986	.020	.892	-.051	.108	-.364	.504	-.111	.693	.003	.821
	**SN**	.027	.625	-.498	.002**	-.291	.119	-.017	.668	-1.642	.018*	-.026	.943	.009	.522
	**N**	.026	.379	-.039	.654	-.040	.695	.052	.015*	-.125	.741	.104	.595	-.005	.541
	**IN**	-.052	.264	.207	.131	.211	.190	-.012	.735	.172	.776	.213	.497	-.007	.583
**DCP**	**Total**	.017	.694	-.279	.021*	-.327	.017*	-.026	.354	-1.196	.017*	-.109	.669	.002	.835
	**ST**	-.007	.921	-.515	.012*	-.411	.089	-.093	.075	-2.001	.027*	-.266	.573	.025	.188
	**T**	-.001	.990	-.041	.818	-.186	.380	.011	.813	-.593	.454	.263	.522	.010	.555
	**IT**	-.082	.248	.093	.641	-.120	.593	-.030	.569	1.112	.191	-.194	.622	.001	.947
	**S**	.054	.507	-.631	.007**	-.538	.048*	-.038	.520	-2.521	.011*	-.092	.860	.001	.955
	**C**	-.038	.473	-.153	.315	-.280	.115	-.019	.628	-.697	.291	.218	.522	-.001	.943
	**I**	.007	.920	-.163	.432	-.211	.373	-.109	.031	-.770	.376	-.522	.243	-.011	.548
	**SN**	.109	.167	-.685	.003**	-.582	.030*	.022	.706	-2.221	.027*	-.125	.812	-.002	.910
	**N**	.152	.113	-.184	.234	-.406	.024*	.045	.256	-.555	.411	-.429	.218	-.030	.028*
	**IN**	-.057	.375	.155	.405	.134	.540	-.064	.177	.118	.886	-.165	.698	-.015	.361
**CC**	**Total**	-.041	.132	-.052	.512	.009	.921	-.024	.193	-.071	.827	-.006	.969	.009	.208
	**ST**	-.053	.358	-.261	.111	-.201	.303	-.100	.015*	-.835	.248	-.304	.417	.021	.167
	**T**	-.104	.014*	.191	.113	.135	.341	.026	.407	.678	.190	.306	.253	.015	.170
	**IT**	-.091	.037*	.136	.275	.169	.225	.019	.555	1.210	.240	.239	.335	.018	.132
	**S**	.047	.315	-.316	.017*	-.217	.166	-.027	.435	-1.149	.047*	-.078	.796	-.003	.805
	**C**	-.024	.721	-.153	.537	-.230	.246	-.038	.402	1.092	.137	-.601	.118	-.024	.165
	**I**	-.004	.919	-.074	.511	-.035	.788	-.026	.358	-.109	.824	-.286	.252	.004	.705
	**SN**	.052	.284	-.380	.005	-.234	.151	-.016	.654	-1.534	.010**	.226	.479	-.004	.776
	**N**	.013	.613	-.066	.384	.058	.517	.028	.140	-.323	.328	.030	.860	-.008	.275
	**IN**	-.020	.572	.170	.092	.244	.369	-.010	.710	.572	.199	.014	.950	-.007	.474
**MLCV**	**Total**	-.037	.000**	.029	.552	-.050	.368	-.009	.445	.168	.404	.012	.904	.004	.374
	**ST**	-.033	.056	.031	.526	.029	.621	-.011	.370	.048	.823	-.158	.151	-.009	.046*
	**T**	-.080	.000**	.035	.617	.066	.419	-.003	.881	.183	.544	-.081	.603	-.002	.717
	**IT**	-.058	.001**	.035	.477	-.034	.538	-.017	.178	.279	.177	-.087	.366	-.003	.539
	**S**	-.027	.096	.001	.982	-.009	.869	-.026	.028*	-.027	.899	.134	.217	.003	.428
	**C**	-.032	.012*	.088	.015*	.016	.718	-.012	.214	.296	.063	.059	.479	-.003	.389
	**I**	-.027	.048*	.040	.329	-.001	.985	-.008	.440	.004	.982	-.029	.747	-.003	.435
	**SN**	-.064	.027*	.026	.784	-.080	.390	-.005	.826	.318	.356	.039	.825	.004	.583
	**N**	-.166	.000**	.056	.695	-.205	.219	.004	.918	1.133	.063	.240	.450	.014	.273
	IN	-.237	.000**	.081	.645	-.150	.450	-.008	.850	.707	.340	.299	.432	.029	.075

Statistically significant values are shown with */**P<0.05 is marked by *P<0.01 is marked by **. ES: effect size (%). FBG, fasting blood-glucose; FINS, fasting insulin; FCP, fasting C-peptide; HbA1c, glycosylated hemoglobin type A1c; eGFR, estimated glomerular filtration rate; VD, vessel density; SCP, superficial capillary plexus; DCP, deep capillary plexus; CC, choriocapillaris; MLCV, mid-to-large choroidal vessel.

## Discussion

DR is one of the most common and damaging microvascular complications of diabetes and a common eye disease leading to blindness, that will eventually develop in almost all patients with diabetes (20). Previous studies have shown that the fundus changes in DR and DC occur first in the midperipheral region (7, 21). Therefore, in this study, 12 mm×12 mm midperipheral fundus images were scanned by WSS-OCTA and the changes in fundus thickness/VD in preclinical and early DR and their correlation with laboratory indices were investigated by quantitative data.

Traditional OCTA is insensitive to imaging blood flow in the choroidal Sattler and Haller layers. TowardPi has a broader scanning range and has developed a three-dimensional spatial recognition algorithm, which identifies OCT signals with differences between large vessels and the stroma by a threshold segmentation algorithm, and then achieved the morphological reconstruction of MLCV (22). As it is supplemented with various signal enhancement and pseudo-signal elimination techniques, the retinal vessels cast less shadow, which in turn results in clearer MLCV layer images and more stable data. Compared with the control group, the decrease in MLCV VD in the NDR group was present in the N, IN, and I regions of the midperiphery of the fundus, whereas no significant difference in MLCV VD was observed in the patients of the NPDR group. Tan et al. showed that the CVI was significantly lower in NDR group than that in control group, which is consistent with the results of the present study, suggesting that the choroidal VD in T2DM patients was reduced before the onset of DR (23). The retinal vasculature lacks autonomic innervation, whereas the choroidal circulation is innervated by sympathetic and parasympathetic nerves (24, 25). We speculate that the decrease in choroidal blood flow in NDR patients may be due to diabetes-induced neurological disorders and vasculopathy, or both. Although choroidal blood flow was normalized in patients with mild NPDR with their own neuromodulation, this does not mean that the choroidal vasculature is not pathologically altered. Improving choroidal blood flow and its adaptive regulation may be a viable therapeutic strategy (26).

Grunwald et al. showed that hemodynamic changes were an early marker of diabetic retinopathy (27). We found that SCP VD (IT, C, I) and DCP VD (T, IT,I) was significantly decreased in the NPDR group compared with the control group, which is consistent with the results of previous studies (27). Of particular note are the IT and I regions, where superficial and deep retinal VD co-varies, and which may be the regions primarily affected in retinal lesions. A study showed that in the peripheral region of the diabetic cohort fundus, the temporal area of the SCP had the lowest perfusion and the inferior sector of the DCP had less perfusion, which may be related to the presence of the temporal watershed of the major temporal vascular arcades (28). In the clinical practice of performing fluorescein fundus angiography, we found that the infra-temporal retinal artery was the first to fill and the last to empty, which corresponds to the IT and I areas in this study. Therefore, we hypothesize that hyperglycemia may cause the inferior temporal retinal artery to be the first to decrease in perfusion.

In addition, we did not find a decrease in VD of SCP or DCP in the NDR group, nor did the VD of CC appear statistically different between groups. However, Cao and colleagues reported that they found changes in SCP, DCP, and CC within a 6-mm × 6-mm area centered on the orbit in NDR patients, which contributed to the detection of preclinical DR (29). Another study found that the VD of the SCP was significantly higher in the NDR group than that in control group (30). Results that are inconsistent with previous studies need to be confirmed in further scientific studies in the future.

In the present study, IRT (ST, IT) and ORT (ST, N) were found increased in the NPDR group compared with controls. Hirotaka et al. found that the retinal capillary perfusion zone (PA-NPDR) was significantly thicker in DR compared with that in non-diabetic retinopathy perfusion zone (PA-NDR), which may be due to leakage of vascular tissue in retinal layers (31). In addition, significantly reduced CT was observed in seven mid-peripheral regions (ST, T, IT, S, I, SN, IN) in NDR patients, but most of these regions were not significantly different in the NPDR group compared with the normal group. A cross-sectional study of 100 NDR eyes by Gupta et al. showed a significant reduction in CT in the central fovea and peripheral area of the macula compared to controls, which is consistent with our findings (32). CT changes may be associated with MLCV filling. As choroidal vasodilation and leakage lead to tissue edema, vascular stiffness increases (33), which may explain the CT rebound in the NPDR group.

We performed a correlation analysis between thickness/VD and laboratory indices. FBG was positively correlated with ORT of the mid-peripheral regions and negatively correlated with VD of part regions in the DCP layer. A number of reports suggested that FBG is an independent risk factor for the occurrence of DR (34). Elevated FBG in diabetic patients increases hemoglobin glycosylation and increased glycosylation of hemoglobin increases its affinity for oxygen, therefore, preventing its release at the tissue inducing hypoxia and oxidative stress (OS), which further leads to a decrease in VD (35).

In patients with T2DM, BMI was positively correlated with ORT and CT in some regions, and negatively correlated with VD in mid-peripheral regions in all layers. Hammes et al. found a significantly increased risk of retinopathy in the obese subgroup by univariate analysis (36). Another study similarly showed a higher incidence of ocular complications in T2DM patients with higher BMI (37). These studies support our results. Therefore, controlling BMI of T2DM patients to normal levels is important to protect retinal structure and blood flow.

In the present study, eGFR was positively correlated with IRT and CT in some regions, and negatively correlated with VD of DCP and MLCV layers in mid-peripheral regions. However, some studies had shown that the occurrence of DR is associated with a decrease in eGFR, which might be that a large numbers of severe NPDR or PDR patients were included in their studies (38, 39). Another study showed an increased trend of eGFR in patients with early T2DM (40), which is consistent with the trend of our research. In the early stage of diabetes, glomerular perfusion pressure increases with the dilatation of renal afferent glomerular arterioles, resulting in the decrease of creatinine and the increase of eGFR. We hypothesized that the increased renal blood may affect the distribution of blood throughout the body and lead to the decrease of blood in the fundus, which may be one of the reasons for the decrease of fundus VD.

The research is limited in several ways: 1) This study collected outpatients and inpatients with T2DM, most of whom were between 40 and 60 years old, hence there was a lack of observation of younger or older diabetics. Small sample size and single-center study design were also limitations. 2) We gathered binocular data from several participants. And generalized linear mixed model was used to diminish the binocular interaction. Subsequent prospective studies with strictly monocular data could further validate our results. 3) No abnormalities were found in the central macular area and no further partitioning study was conducted. This study focused on the midperipheral fundus.

## Conclusion

Structural and blood flow changes in the choroid appear before the onset of DR and precede changes in the retinal microcirculation, and MLCV thickness/VD is a more sensitive imaging biomarker for clinical detection of DR. Choriocapillaris are not affected in preclinical DR and early DR, therefore more attention could be paid to the MLCV. FBG, BMI and eGFR levels have relationship with thickness/VD in fundus of T2DM patients, which may be associated with the occurrence and development of DR. WSS-OCTA enables large-scale non-invasive visual screening and follow-up of the retinal and choroidal vasculature in DR patients, which provides a new strategy for the prevention and monitoring of DR in patients with T2DM.

## Data availability statement

The original contributions presented in the study are included in the article/**Supplementary Material**. Further inquiries can be directed to the corresponding author.

## Ethics statement

The studies involving human participants were reviewed and approved by Ethics Committee of Qilu Hospital of Shandong University. The patients/participants provided their written informed consent to participate in this study.

## Author contributions

ZQ and YS: study concept and design; ZQ, JZ, XY, and WW: biological material collection and data acquisition; FF and YS: data analysis and interpretation; ZQ and YZ: overall study supervision; and ZQ and YS: drafting of the manuscript. ZQ, YS, and FF also reviewed the underlying data. All authors contributed to the article and approved the submitted version.
