# Supplementary material for: Analysis of retinal and choroidal characteristics in patients with early diabetic retinopathy using WSS-OCTA
Source: Front Endocrinol (Lausanne). 2023 May 24;14:1184717. doi: 10.3389/fendo.2023.1184717 (PMC10244727; doi:10.3389/fendo.2023.1184717)
Supplement: Supplementary file 1 [file Table_1.docx]

**Table S1. Correlation analysis between right eye thickness and clinical physiological indexes in T2DM**

| **Layer** | **Region** | **Age** | | **BMI** | | **FBG** | | **FINS** | | **FCP** | | **HbA1c** | | **eGFR** | |
| --- | --- | --- | --- | --- | --- | --- | --- | --- | --- | --- | --- | --- | --- | --- | --- |
|  |  | **ES** | **P** | **ES** | **P** | **ES** | **P** | **ES** | **P** | **ES** | **P** | **ES** | **P** | **ES** | **P** |
| **IRT** | **Total** | -.274 | 0.006** | .199 | 0.492 | .478 | 0.122 | -.018 | 0.796 | .156 | 0.904 | .641 | 0.254 | .054 | 0.046* |
|  | **ST** | -.193 | 0.023* | .143 | 0.583 | .271 | 0.349 | .080 | 0.137 | .869 | 0.434 | .869 | 0.044* | .026 | 0.245 |
|  | **T** | -.170 | 0.055 | .273 | 0.313 | .273 | 0.366 | .006 | 0.915 | .504 | 0.663 | .079 | 0.896 | .034 | 0.147 |
|  | **IT** | -.156 | 0.030* | .015 | 0.944 | .184 | 0.446 | .013 | 0.807 | .476 | 0.607 | .298 | 0.504 | .041 | 0.033* |
|  | **S** | -.492 | 0.000** | .344 | 0.414 | .517 | 0.270 | -.021 | 0.835 | 1.415 | 0.432 | 1.032 | 0.274 | .065 | 0.085 |
|  | **C** | -.368 | 0.001** | .446 | 0.190 | .519 | 0.170 | -.054 | 0.494 | 1.198 | 0.411 | .234 | 0.761 | .083 | 0.003** |
|  | **I** | -.265 | 0.003** | .160 | 0.542 | .222 | 0.467 | -.009 | 0.890 | .098 | 0.931 | .417 | 0.473 | .061 | 0.009** |
|  | **SN** | -.303 | 0.064 | -.082 | 0.868 | .501 | 0.361 | -.143 | 0.202 | -1.488 | 0.480 | .499 | 0.652 | .044 | 0.315 |
|  | **N** | -.691 | 0.000** | .211 | 0.701 | .876 | 0.150 | -.162 | 0.207 | -.841 | 0.720 | .939 | 0.445 | .143 | 0.003** |
|  | **IN** | -.186 | 0.053 | .224 | 0.437 | .280 | 0.383 | .006 | 0.925 | -.038 | 0.976 | .802 | 0.214 | .037 | 0.146 |
| **ORT** | **Total** | -.221 | 0.007** | .544 | 0.025* | .559 | 0.03* | -.058 | 0.311 | -.414 | 0.710 | .206 | 0.663 | .023 | 0.323 |
|  | **ST** | -.432 | 0.000** | .559 | 0.044* | .607 | 0.051 | -.083 | 0.092 | .991 | 0.410 | -.137 | 0.822 | .020 | 0.404 |
|  | **T** | -.268 | 0.002** | .390 | 0.249 | .684 | 0.076 | -.058 | 0.315 | -.368 | 0.804 | .498 | 0.460 | .073 | 0.000** |
|  | **IT** | -.212 | 0.018* | .497 | 0.065 | .612 | 0.041* | -.056 | 0.365 | -.394 | 0.735 | .109 | 0.856 | .011 | 0.645 |
|  | **S** | -.118 | 0.153 | .370 | 0.000** | .552 | 0.121 | .017 | 0.010* | -.149 | 0.899 | .296 | 0.056 | .002 | 0.846 |
|  | **C** | -.192 | 0.136 | .497 | 0.213 | .185 | 0.682 | -.030 | 0.715 | -.855 | 0.619 | -.107 | 0.902 | .060 | 0.066 |
|  | **I** | -.226 | 0.004** | .358 | 0.125 | .387 | 0.150 | -.049 | 0.384 | .109 | 0.913 | .013 | 0.980 | .038 | 0.065 |
|  | **SN** | -.210 | 0.057 | .480 | 0.149 | .867 | 0.018* | -.076 | 0.333 | -.659 | 0.644 | .797 | 0.275 | -.005 | 0.864 |
|  | **N** | -.331 | 0.033* | .339 | 0.483 | 1.932 | 0.000** | -.117 | 0.263 | .606 | 0.771 | 3.222 | 0.000** | .061 | 0.123 |
|  | **IN** | -.266 | 0.012* | .346 | 0.279 | .346 | 0.185 | -.051 | 0.504 | -1.190 | 0.384 | -.040 | 0.956 | .024 | 0.413 |
| **CT** | **Total** | -2.959 | 0.000** | 4.019 | 0.037* | -.489 | 0.812 | -.581 | 0.109 | 9.264 | 0.281 | -.016 | 0.996 | .438 | 0.011* |
|  | **ST** | -2.825 | 0.000** | 4.506 | 0.060 | 1.873 | 0.500 | .750 | 0.000** | 18.646 | 0.075 | 1.736 | 0.728 | -.143 | 0.478 |
|  | **T** | -1.703 | 0.013* | 3.250 | 0.139 | 1.170 | 0.660 | -.167 | 0.648 | 8.981 | 0.369 | 3.830 | 0.306 | .196 | 0.176 |
|  | **IT** | -1.584 | 0.038* | 4.697 | 0.039* | 1.951 | 0.447 | -.499 | 0.360 | 13.115 | 0.181 | -2.404 | 0.643 | -.001 | 0.995 |
|  | **S** | -3.749 | 0.000** | 5.406 | 0.053 | 2.747 | 0.382 | -.468 | 0.484 | 24.917 | 0.036* | -2.575 | 0.685 | .052 | 0.837 |
|  | **C** | -2.857 | 0.004** | 2.023 | 0.507 | -.311 | 0.927 | -.851 | 0.236 | 15.841 | 0.221 | -1.535 | 0.823 | .293 | 0.282 |
|  | **I** | -3.370 | 0.000** | 3.422 | 0.060 | 1.959 | 0.353 | -.496 | 0.244 | 10.430 | 0.170 | 2.049 | 0.595 | .523 | 0.002** |
|  | **SN** | -2.417 | 0.005** | 4.998 | 0.054 | -.486 | 0.868 | -.359 | 0.563 | 17.745 | 0.110 | -2.054 | 0.727 | .183 | 0.435 |
|  | **N** | -1.460 | 0.069 | 1.552 | 0.521 | -1.713 | 0.524 | -.664 | 0.224 | 13.891 | 0.176 | 1.167 | 0.830 | .326 | 0.131 |
|  | **IN** | -1.738 | 0.003** | 2.404 | 0.179 | -1.967 | 0.325 | -.210 | 0.621 | 8.954 | 0.242 | -1.048 | 0.795 | .257 | 0.109 |

Statistically significant values are shown with */**, P＜0.05 is marked by *, P＜0.01 is marked by **. ES: effect size (um). FBG, fasting blood-glucose; FINS, fasting insulin; FCP, fasting C-peptide; HbA1c, glycosylated hemoglobin type A1c; eGFR, estimated glomerular filtration rate; IRT, inner retinal thickness; ORT, outer retinal thickness; CT, choroidal thickness.
