# Supplementary material for: Analysis of retinal and choroidal characteristics in patients with early diabetic retinopathy using WSS-OCTA
Source: Front Endocrinol (Lausanne). 2023 May 24;14:1184717. doi: 10.3389/fendo.2023.1184717 (PMC10244727; doi:10.3389/fendo.2023.1184717)
Supplement: Supplementary file 2 [file Table_2.docx]

**Table S2. Correlation analysis between right eye VD and clinical physiological indexes in T2DM**

| **Layer** | **Region** | **Age** | | **BMI** | | **FBG** | | **FINS** | | **FCP** | | **HbA1c** | | **eGFR** | |
| --- | --- | --- | --- | --- | --- | --- | --- | --- | --- | --- | --- | --- | --- | --- | --- |
|  |  | **ES** | **P** | **ES** | **P** | **ES** | **P** | **ES** | **P** | **ES** | **P** | **ES** | **P** | **ES** | **P** |
| **SCP** | **Total** | -.006 | 0.848 | -.212 | 0.021* | -.112 | 0.254 | -.019 | 0.392 | -.548 | 0.185 | -.105 | 0.558 | .011 | 0.210 |
|  | **ST** | -.006 | 0.922 | -.391 | 0.041* | -.232 | 0.281 | -.020 | 0.666 | -.573 | 0.488 | -.104 | 0.811 | .031 | 0.074 |
|  | **T** | .124 | 0.002** | .010 | 0.956 | -.170 | 0.429 | .054 | 0.007** | .954 | 0.242 | .315 | 0.406 | .004 | 0.796 |
|  | **IT** | .032 | 0.637 | -.362 | 0.074 | .135 | 0.554 | .019 | 0.691 | .316 | 0.718 | .436 | 0.340 | .044 | 0.014 |
|  | **S** | -.084 | 0.180 | -.532 | 0.003** | -.104 | 0.619 | -.070 | 0.109 | -1.523 | 0.054 | .306 | 0.466 | .037 | 0.026 |
|  | **C** | .006 | 0.859 | .094 | 0.445 | -.134 | 0.414 | .017 | 0.008** | -.278 | 0.646 | .324 | 0.017* | .003 | 0.778 |
|  | **I** | -.067 | 0.120 | .002 | 0.986 | .025 | 0.869 | -.055 | 0.060 | -.423 | 0.436 | -.045 | 0.871 | .010 | 0.403 |
|  | **SN** | .016 | 0.818 | -.862 | 0.000** | -.310 | 0.180 | -.070 | 0.152 | -2.410 | 0.006** | -.451 | 0.335 | .028 | 0.137 |
|  | **N** | .016 | 0.589 | -.037 | 0.675 | -.090 | 0.366 | .050 | 0.005** | -.501 | 0.243 | -.034 | 0.866 | -.004 | 0.611 |
|  | **IN** | -.034 | 0.564 | .647 | 0.000** | .476 | 0.033 | .031 | 0.014* | -.137 | 0.872 | .535 | 0.034* | -.013 | 0.383 |
| **DCP** | **Total** | -.003 | 0.944 | -.324 | 0.013* | -.275 | 0.053 | -.049 | 0.120 | -.712 | 0.237 | -.167 | 0.517 | .008 | 0.524 |
|  | **ST** | -.071 | 0.393 | -.422 | 0.092 | -.242 | 0.386 | -.102 | 0.027* | -.834 | 0.437 | .257 | 0.646 | .039 | 0.079 |
|  | **T** | -.060 | 0.471 | .057 | 0.825 | -.242 | 0.404 | .044 | 0.408 | 1.216 | 0.272 | 1.018 | 0.051 | .036 | 0.084 |
|  | **IT** | -.072 | 0.407 | -.380 | 0.139 | .229 | 0.425 | -.013 | 0.832 | .538 | 0.625 | .896 | 0.119 | .054 | 0.018* |
|  | **S** | -.071 | 0.483 | -.867 | 0.003** | -.506 | 0.129 | -.147 | 0.019* | -2.451 | 0.054 | .108 | 0.873 | .039 | 0.142 |
|  | **C** | -.119 | 0.100 | -.125 | 0.582 | -.322 | 0.206 | -.030 | 0.520 | -.487 | 0.618 | .671 | 0.159 | .026 | 0.163 |
|  | **I** | -.001 | 0.986 | -.161 | 0.427 | -.208 | 0.377 | -.102 | 0.030* | -.941 | 0.273 | -.311 | 0.476 | -.001 | 0.961 |
|  | **SN** | .106 | 0.277 | -1.227 | 0.000** | -.792 | 0.013* | -.131 | 0.020* | -3.290 | 0.007** | -1.023 | 0.113 | .024 | 0.352 |
|  | **N** | .179 | 0.022* | -.271 | 0.122 | -.493 | 0.010* | .043 | 0.232 | -1.389 | 0.062 | -.515 | 0.188 | -.029 | 0.064 |
|  | **IN** | -.038 | 0.651 | .584 | 0.020* | .424 | 0.140 | .006 | 0.908 | -.002 | 0.999 | .000 | 1.000 | -.042 | 0.035* |
| **CC** | **Total** | -.032 | 0.278 | -.058 | 0.505 | -.018 | 0.843 | -.025 | 0.229 | .105 | 0.786 | -.126 | 0.445 | .007 | 0.406 |
|  | **ST** | -.092 | 0.248 | -.175 | 0.463 | -.048 | 0.857 | -.048 | 0.083 | -.256 | 0.801 | -.177 | 0.741 | .026 | 0.214 |
|  | **T** | -.104 | 0.070 | .035 | 0.078 | .069 | 0.723 | .044 | 0.283 | 1.409 | 0.057 | .271 | 0.489 | .020 | 0.201 |
|  | **IT** | -.090 | 0.142 | .064 | 0.736 | .314 | 0.134 | .021 | 0.590 | 1.073 | 0.181 | .697 | 0.077 | .040 | 0.010* |
|  | **S** | -.027 | 0.641 | -.441 | 0.011* | -.167 | 0.395 | -.078 | 0.009** | -1.073 | 0.152 | -.091 | 0.816 | .020 | 0.188 |
|  | **C** | -.037 | 0.497 | .381 | 0.027* | .019 | 0.944 | -.004 | 0.857 | 1.797 | 0.074 | .407 | 0.019* | .006 | 0.623 |
|  | **I** | -.006 | 0.882 | -.072 | 0.521 | -.034 | 0.795 | -.031 | 0.268 | -.108 | 0.826 | -.266 | 0.286 | .005 | 0.618 |
|  | **SN** | .057 | 0.316 | -.731 | 0.000** | -.226 | 0.234 | -.083 | 0.004** | -2.331 | 0.001** | -.196 | 0.608 | .010 | 0.522 |
|  | **N** | .017 | 0.603 | -.046 | 0.641 | .001 | 0.995 | .039 | 0.059 | -.593 | 0.158 | -.094 | 0.671 | -.011 | 0.225 |
|  | **IN** | -.020 | 0.700 | .357 | 0.025* | .373 | 0.040* | .043 | 0.016* | .257 | 0.714 | .140 | 0.677 | -.004 | 0.784 |
| **MLCV** | **Total** | -.087 | 0.000** | .079 | 0.275 | -.110 | 0.152 | -.016 | 0.309 | .528 | 0.100 | -.112 | 0.395 | .002 | 0.747 |
|  | **ST** | -.039 | 0.141 | .107 | 0.174 | .027 | 0.757 | -.012 | 0.511 | .284 | 0.398 | -.276 | 0.116 | -.016 | 0.021* |
|  | **T** | -.076 | 0.003** | .101 | 0.330 | .080 | 0.519 | -.008 | 0.555 | .431 | 0.354 | -.009 | 0.961 | .015 | 0.000** |
|  | **IT** | -.078 | 0.002** | .107 | 0.177 | .036 | 0.689 | -.009 | 0.560 | .428 | 0.213 | .026 | 0.874 | .002 | 0.786 |
|  | **S** | -.021 | 0.417 | .112 | 0.153 | -.083 | 0.375 | -.048 | 0.011* | .237 | 0.506 | .262 | 0.027* | .003 | 0.635 |
|  | **C** | -.047 | 0.009** | .102 | 0.057 | -.008 | 0.898 | -.025 | 0.037* | .384 | 0.094 | .045 | 0.714 | -.002 | 0.712 |
|  | **I** | -.028 | 0.039* | .040 | 0.327 | -.002 | 0.965 | -.010 | 0.330 | -.015 | 0.934 | -.025 | 0.785 | -.003 | 0.481 |
|  | **SN** | 0.000 | 0.999 | 0.000 | 0.999 | 0.000 | 0.996 | 0.000 | 1.000 | 0.000 | 0.997 | 0.000 | 1.000 | 0.000 | 1.000 |
|  | **N** | -.171 | 0.004** | .029 | 0.873 | -.266 | 0.196 | .054 | 0.002** | 1.239 | 0.114 | 0.498 | 0.189 | .014 | 0.382 |
|  | **IN** | -.279 | 0.000** | .157 | 0.483 | -.190 | 0.447 | -.026 | 0.583 | 1.084 | 0.254 | .293 | 0.547 | .052 | 0.002** |

Statistically significant values are shown with */**, P＜0.05 is marked by *, P＜0.01 is marked by **. ES: effect size (%). FBG, fasting blood-glucose; FINS, fasting insulin; FCP, fasting C-peptide; HbA1c, glycosylated hemoglobin type A1c; eGFR, estimated glomerular filtration rate; VD, vessel density; SCP, superficial capillary plexus; DCP, deep capillary plexus; CC, choriocapillaris; MLCV, mid-to-large choroidal vessel.
