# Supplementary material for: Analysis of retinal and choroidal characteristics in patients with early diabetic retinopathy using WSS-OCTA
Source: Front Endocrinol (Lausanne). 2023 May 24;14:1184717. doi: 10.3389/fendo.2023.1184717 (PMC10244727; doi:10.3389/fendo.2023.1184717)
Supplement: Supplementary file 3 [file Table_3.docx]

**Table S3. Correlation analysis between left eye VD and clinical physiological indexes in T2DM**

| **Layer** | **Region** | **Age** | | **BMI** | | **FBG** | | **FINS** | | **FCP** | | **HbA1c** | | **eGFR** | |
| --- | --- | --- | --- | --- | --- | --- | --- | --- | --- | --- | --- | --- | --- | --- | --- |
|  |  | **ES** | **P** | **ES** | **P** | **ES** | **P** | **ES** | **P** | **ES** | **P** | **ES** | **P** | **ES** | **P** |
| **SCP** | **Total** | .011 | 0.644 | -.045 | 0.514 | -.108 | 0.181 | -.008 | 0.671 | -.544 | 0.060 | -.103 | 0.465 | -.006 | 0.375 |
|  | **ST** | .044 | 0.490 | -.598 | 0.001** | -.322 | 0.164 | -.049 | 0.332 | -2.366 | 0.004** | -.388 | 0.365 | .007 | 0.656 |
|  | **T** | .095 | 0.140 | -.099 | 0.592 | -.090 | 0.696 | -.001 | 0.978 | -1.574 | 0.058 | -.197 | 0.648 | -.016 | 0.341 |
|  | **IT** | -.097 | 0.216 | .585 | 0.008** | .107 | 0.704 | .023 | 0.718 | 1.022 | 0.318 | -.074 | 0.890 | -.026 | 0.195 |
|  | **S** | .078 | 0.215 | -.245 | 0.174 | -.237 | 0.290 | -.009 | 0.854 | -1.896 | 0.019* | -.084 | 0.842 | -.006 | 0.704 |
|  | **C** | .097 | 0.033* | -.023 | 0.858 | -.082 | 0.614 | -.003 | 0.914 | -.630 | 0.289 | -.232 | 0.450 | -.025 | 0.030* |
|  | **I** | -.089 | 0.108 | .118 | 0.462 | -.099 | 0.620 | -.064 | 0.139 | -.305 | 0.676 | -.435 | 0.242 | .008 | 0.581 |
|  | **SN** | .030 | 0.690 | -.186 | 0.382 | -.324 | 0.219 | .013 | 0.820 | -.994 | 0.304 | .139 | 0.782 | -.006 | 0.751 |
|  | **N** | .031 | 0.474 | -.047 | 0.704 | -.021 | 0.894 | .049 | 0.147 | .169 | 0.763 | .064 | 0.825 | -.005 | 0.677 |
|  | **IN** | -.043 | 0.470 | -.093 | 0.588 | -.104 | 0.626 | -.025 | 0.605 | .382 | 0.625 | -.011 | 0.978 | .007 | 0.644 |
| **DCP** | **Total** | .037 | 0.407 | -.096 | 0.435 | -.285 | 0.044* | -.019 | 0.586 | -.740 | 0.150 | -.349 | 0.155 | -.016 | 0.166 |
|  | **ST** | .068 | 0.414 | -.619 | 0.008 | -.496 | 0.093 | -.069 | 0.300 | -2.963 | 0.005** | -.308 | 0.583 | .009 | 0.690 |
|  | **T** | .056 | 0.456 | -.091 | 0.677 | -.066 | 0.808 | -.007 | 0.908 | -2.414 | 0.013* | .009 | 0.986 | -.008 | 0.677 |
|  | **IT** | -.152 | 0.135 | .756 | 0.008 | .016 | 0.965 | .014 | 0.861 | 1.016 | 0.446 | .033 | 0.962 | -.026 | 0.312 |
|  | **S** | .159 | 0.131 | -.406 | 0.180 | -.419 | 0.265 | .055 | 0.512 | -2.625 | 0.055 | -.354 | 0.619 | -.028 | 0.310 |
|  | **C** | .032 | 0.580 | -.110 | 0.507 | -.159 | 0.440 | .005 | 0.908 | .005 | 0.908 | -.062 | 0.847 | -.018 | 0.229 |
|  | **I** | -.017 | 0.843 | .018 | 0.943 | -.303 | 0.324 | -.164 | 0.013* | -.816 | 0.468 | -1.044 | 0.068 | -.003 | 0.899 |
|  | **SN** | .089 | 0.351 | -.225 | 0.412 | -.439 | 0.195 | .084 | 0.263 | -1.393 | 0.263 | .247 | 0.700 | -.020 | 0.410 |
|  | **N** | .120 | 0.123 | -.129 | 0.566 | -.337 | 0.223 | .058 | 0.346 | .222 | 0.827 | -.536 | 0.306 | -.032 | 0.114 |
|  | **IN** | -.057 | 0.526 | -.224 | 0.384 | -.211 | 0.509 | -.119 | 0.092 | .292 | 0.803 | -.453 | 0.453 | .008 | 0.717 |
| **CC** | **Total** | .001 | 0.963 | -.029 | 0.593 | .006 | 0.927 | .000 | 0.985 | -.322 | 0.156 | .085 | 0.441 | -.003 | 0.542 |
|  | **ST** | .003 | 0.949 | -.370 | 0.006** | -.263 | 0.122 | -.069 | 0.067 | -1.265 | 0.040* | -.017 | 0.957 | .015 | 0.227 |
|  | **T** | -.084 | 0.023* | .121 | 0.260 | .219 | 0.098 | .003 | 0.914 | .087 | 0.856 | .440 | 0.078 | .005 | 0.629 |
|  | **IT** | -.136 | 0.038* | .257 | 0.175 | .254 | 0.280 | .039 | 0.458 | 1.304 | 0.129 | .540 | 0.224 | .009 | 0.605 |
|  | **S** | .092 | 0.109 | -.193 | 0.259 | -.295 | 0.159 | .021 | 0.626 | -1.181 | 0.126 | -.344 | 0.345 | -.021 | 0.127 |
|  | **C** | .074 | 0.070 | -.067 | 0.574 | .023 | 0.878 | .054 | 0.097 | .264 | 0.622 | -.166 | 0.548 | -.026 | 0.014* |
|  | **I** | .017 | 0.710 | -.078 | 0.541 | -.140 | 0.376 | -.032 | 0.359 | -.321 | 0.581 | -.606 | 0.041* | .006 | 0.595 |
|  | **SN** | .036 | 0.563 | -.077 | 0.671 | -.301 | 0.177 | -.023 | 0.646 | -.869 | 0.288 | .309 | 0.464 | -.013 | 0.413 |
|  | **N** | .006 | 0.869 | -.091 | 0.370 | .095 | 0.449 | .017 | 0.526 | -.118 | 0.797 | .149 | 0.513 | -.006 | 0.499 |
|  | **IN** | -.007 | 0.878 | .060 | 0.627 | .112 | 0.468 | -.033 | 0.332 | .897 | 0.110 | .088 | 0.764 | -.007 | 0.534 |
| **MLCV** | **Total** | -.067 | 0.000** | .029 | 0.601 | -.065 | 0.312 | -.010 | 0.533 | .284 | 0.220 | -.056 | 0.610 | -.001 | 0.886 |
|  | **ST** | -.025 | 0.101 | -.007 | 0.872 | .041 | 0.456 | -.010 | 0.427 | -.114 | 0.569 | -.037 | 0.724 | -.004 | 0.303 |
|  | **T** | -.062 | 0.004** | .016 | 0.807 | .073 | 0.351 | .008 | 0.646 | -.039 | 0.891 | -.031 | 0.835 | .000 | 0.968 |
|  | **IT** | -.048 | 0.009* | .021 | 0.696 | -.006 | 0.926 | -.002 | 0.914 | -.120 | 0.618 | .054 | 0.662 | -.002 | 0.731 |
|  | **S** | -.022 | 0.143 | -.050 | 0.246 | .071 | 0.184 | .007 | 0.533 | -.290 | 0.138 | .099 | 0.324 | .001 | 0.776 |
|  | **C** | -.021 | 0.159 | .081 | 0.052 | .043 | 0.412 | .004 | 0.741 | .227 | 0.236 | .084 | 0.396 | -.004 | 0.296 |
|  | **I** | -.022 | 0.147 | .053 | 0.224 | .007 | 0.905 | -.011 | 0.369 | -.030 | 0.881 | -.023 | 0.827 | -.004 | 0.346 |
|  | **SN** | -.070 | 0.028* | -.003 | 0.974 | -.077 | 0.534 | -.037 | 0.083 | .219 | 0.628 | .246 | 0.201 | .013 | 0.013* |
|  | **N** | -.149 | 0.015* | .184 | 0.316 | -.131 | 0.561 | .044 | 0.309 | 1.596 | 0.052 | -.356 | 0.342 | -.002 | 0.916 |
|  | **IN** | -.195 | 0.005** | .080 | 0.695 | -.167 | 0.511 | -.006 | 0.914 | .263 | 0.778 | .393 | 0.412 | .013 | 0.477 |

Statistically significant values are shown with */**, P＜0.05 is marked by *, P＜0.01 is marked by **. ES: effect size (%). FBG, fasting blood-glucose; FINS, fasting insulin; FCP, fasting C-peptide; HbA1c, glycosylated hemoglobin type A1c; eGFR, estimated glomerular filtration rate; VD, vessel density; SCP, superficial capillary plexus; DCP, deep capillary plexus; CC, choriocapillaris; MLCV, mid-to-large choroidal vessel.
