# Supplementary material for: Analysis of retinal and choroidal characteristics in patients with early diabetic retinopathy using WSS-OCTA
Source: Front Endocrinol (Lausanne). 2023 May 24;14:1184717. doi: 10.3389/fendo.2023.1184717 (PMC10244727; doi:10.3389/fendo.2023.1184717)
Supplement: Supplementary file 4 [file Table_4.docx]

**Table S4. Correlation analysis between left eye thickness and clinical physiological indexes in T2DM**

| **Layer** | **Region** | **Age** | | **BMI** | | **FBG** | | **FINS** | | **FCP** | | **HbA1c** | | **eGFR** | |
| --- | --- | --- | --- | --- | --- | --- | --- | --- | --- | --- | --- | --- | --- | --- | --- |
|  |  | **ES** | **P** | **ES** | **P** | **ES** | **P** | **ES** | **P** | **ES** | **P** | **ES** | **P** | **ES** | **P** |
| **IRT** | **Total** | -.165 | 0.101 | .133 | 0.636 | .034 | 0.917 | -.111 | 0.165 | -1.501 | 0.205 | .500 | 0.387 | .057 | 0.032* |
|  | **ST** | -.028 | 0.771 | .143 | 0.607 | .067 | 0.846 | -.077 | 0.313 | -1.067 | 0.330 | .325 | 0.616 | .029 | 0.254 |
|  | **T** | -.031 | 0.745 | .041 | 0.882 | -.004 | 0.990 | -.104 | 0.169 | -1.522 | 0.181 | .169 | 0.795 | .030 | 0.228 |
|  | **IT** | -.051 | 0.549 | -.200 | 0.412 | .156 | 0.606 | -.026 | 0.702 | -1.485 | 0.145 | .251 | 0.661 | .015 | 0.486 |
|  | **S** | -.268 | 0.057 | .442 | 0.292 | .104 | 0.836 | -.292 | 0.008** | -.828 | 0.642 | .586 | 0.526 | .090 | 0.017* |
|  | **C** | -.249 | 0.063 | .339 | 0.381 | -.061 | 0.900 | -.184 | 0.083 | -2.057 | 0.232 | .996 | 0.272 | .103 | 0.003** |
|  | **I** | -.222 | 0.026* | -.011 | 0.969 | -.011 | 0.969 | .023 | 0.948 | -.021 | 0.790 | -1.096 | 0.394 | .692 | 0.308 |
|  | **SN** | -.403 | 0.008* | .461 | 0.313 | .055 | 0.923 | -.248 | 0.042* | -1.884 | 0.358 | 1.025 | 0.330 | .056 | 0.163 |
|  | **N** | -.454 | 0.007** | -.325 | 0.510 | -.055 | 0.928 | -.186 | 0.166 | -3.252 | 0.144 | 1.430 | 0.211 | .148 | 0.011* |
|  | **IN** | -.041 | 0.723 | -.103 | 0.756 | -.052 | 0.900 | .029 | 0.751 | -1.916 | 0.193 | .921 | 0.234 | .038 | 0.208 |
| **ORT** | **Total** | -.170 | 0.023* | .478 | 0.022* | .420 | 0.085 | -.043 | 0.469 | .558 | 0.532 | .315 | 0.460 | .023 | 0.249 |
|  | **ST** | -.268 | 0.006** | .423 | 0.140 | .445 | 0.211 | -.077 | 0.335 | 1.257 | 0.333 | .335 | 0.620 | .014 | 0.592 |
|  | **T** | -.287 | 0.022* | .395 | 0.277 | .715 | 0.111 | -.095 | 0.345 | 2.226 | 0.173 | .572 | 0.502 | .023 | 0.491 |
|  | **IT** | -.105 | 0.335 | -.303 | 0.332 | .402 | 0.299 | -.033 | 0.706 | -1.469 | 0.298 | .935 | 0.199 | .050 | 0.078 |
|  | **S** | -.079 | 0.435 | .394 | 0.172 | .157 | 0.663 | -.172 | 0.029* | 1.392 | 0.274 | -.429 | 0.527 | .003 | 0.915 |
|  | **C** | -.161 | 0.144 | .571 | 0.069 | -.049 | 0.902 | -.127 | 0.146 | 1.972 | 0.159 | -.845 | 0.254 | .003 | 0.921 |
|  | **I** | -.175 | 0.079 | .083 | 0.773 | .335 | 0.348 | -.068 | 0.389 | .057 | 0.965 | .214 | 0.751 | .056 | 0.029* |
|  | **SN** | -.203 | 0.043* | .645 | 0.025* | .640 | 0.074 | -.089 | 0.269 | .900 | 0.497 | .226 | 0.741 | .017 | 0.522 |
|  | **N** | -.129 | 0.252 | .704 | 0.207 | .656 | 0.098 | .025 | 0.778 | .645 | 0.650 | .370 | 0.624 | .000 | 0.990 |
|  | **IN** | -.217 | 0.036* | .576 | 0.053 | .749 | 0.042* | .003 | 0.972 | .310 | 0.819 | 1.044 | 0.136 | .035 | 0.203 |
| **CT** | **Total** | -2.172 | 0.001** | 3.055 | 0.122 | -3.414 | 0.125 | -.652 | 0.217 | 10.278 | 0.201 | -.266 | 0.936 | .305 | 0.073 |
|  | **ST** | -2.004 | 0.005** | 4.606 | 0.030* | -.512 | 0.847 | -.181 | 0.756 | 12.617 | 0.193 | -6.518 | 0.182 | -.165 | 0.381 |
|  | **T** | -1.823 | 0.018* | 2.511 | 0.265 | -2.775 | 0.320 | .155 | 0.801 | 15.129 | 0.135 | -2.261 | 0.666 | .132 | 0.514 |
|  | **IT** | -2.320 | 0.000** | 3.007 | 0.144 | -.971 | 0.703 | -.343 | 0.515 | 11.313 | 0.226 | .795 | 0.862 | .244 | 0.126 |
|  | **S** | -2.902 | 0.001** | 2.245 | 0.382 | -2.423 | 0.446 | .073 | 0.918 | -.084 | 0.994 | -.378 | 0.950 | .092 | 0.691 |
|  | **C** | -3.063 | 0.001** | 1.560 | 0.577 | -3.292 | 0.341 | -.130 | 0.866 | 10.296 | 0.415 | 3.509 | 0.590 | .364 | 0.147 |
|  | **I** | -2.884 | 0.000** | 2.951 | 0.165 | -.203 | 0.939 | -.096 | 0.870 | 7.775 | 0.422 | 7.584 | 0.125 | .484 | 0.10* |
|  | **SN** | -2.068 | 0.011* | 3.425 | 0.149 | -3.991 | 0.176 | .130 | 0.843 | 14.590 | 0.176 | -1.817 | 0.745 | .187 | 0.387 |
|  | **N** | -2.141 | 0.012* | 1.998 | 0.424 | -3.588 | 0.245 | -.045 | 0.948 | 14.113 | 0.212 | 3.588 | 0.538 | .552 | 0.012* |
|  | **IN** | -1.704 | 0.005** | 1.925 | 0.283 | -2.929 | 0.187 | -.077 | 0.877 | 4.391 | 0.590 | 2.764 | 0.511 | .424 | 0.008** |

Statistically significant values are shown with */**, P＜0.05 is marked by *, P＜0.01 is marked by **. ES: effect size (um). FBG, fasting blood-glucose; FINS, fasting insulin; FCP, fasting C-peptide; HbA1c, glycosylated hemoglobin type A1c; eGFR, estimated glomerular filtration rate; IRT, inner retinal thickness; ORT, outer retinal thickness; CT, choroidal thickness.
