# Supplementary material for: Analysis of retinal and choroidal characteristics in patients with early diabetic retinopathy using WSS-OCTA
Source: Front Endocrinol (Lausanne). 2023 May 24;14:1184717. doi: 10.3389/fendo.2023.1184717 (PMC10244727; doi:10.3389/fendo.2023.1184717)
Supplement: Supplementary file 5 [file Table_5.docx]

**Table S5. Differences in AL, SE, and IOP between groups**

|  | control | NDR | NPDR | P_1_ | P_2_ | P_3_ |
| --- | --- | --- | --- | --- | --- | --- |
| Patients (male) | 20 (9) | 53 (28) | 40 (24) | / | / | / |
| Eyes | 28 | 89 | 58 | / | / | / |
| AL (mm) | 24.713±0.184 | 24.719±0.103 | 24.723±0.130 | 0.979 | 0.850 | 0.823 |
| SE (-D) | 0.806±0.171 | 1.081±0.094 | 0.996±0.117 | 0.161 | 0.361 | 0.555 |
| IOP (mmHg) | 15.984±0.567 | 15.951±0.313 | 16.256±0.390 | 0.960 | 0.693 | 0.525 |

P_1_: control vs. NDR；P_2_: control vs. NPDR；P_3_: NDR vs. NPDR. Control, healthy subjects; NDR, non-diabetic retinopathy; NPDR, non-proliferative diabetic retinopathy; AL, axial length; SE, spherical equivalent; IOP, intraocular pressure.
